# Optical and clinical simulated performance of a new refractive extended depth of focus intraocular lens

**DOI:** 10.1038/s41433-024-03041-0

**Published:** 2024-04-05

**Authors:** Aixa Alarcon, Antonio del Aguila Carrasco, Franck Gounou, Henk Weeber, Carmen Cánovas, Patricia Piers

**Affiliations:** Johnson and Johnson Vision Van Swietenlaan 5, Groningen, 9728 NX The Netherlands

**Keywords:** Outcomes research, Quality of life

## Abstract

**Objectives:**

The purpose of this study is to evaluate the optical and expected clinical performance of a new refractive Extended Depth of Focus (EDF) intraocular lens (IOL), TECNIS PureSee™ IOL, designed to maintain a monofocal-like dysphotopsia profile.

**Methods:**

Simulated visual acuity (sVA) with varying defocus was calculated using the area under the Modulation Transfer Function measured in an average eye model and from computer simulations in eye models with corneal higher-order aberrations. Tolerance to defocus was evaluated using computer simulations of the uncorrected distance sVA under defocus. To evaluate the dysphotopsia profile, halo pictures obtained using an IOL-telescope, as well as simulated images in a realistic eye model under defocus were assessed. The results of the refractive EDF, TECNIS PureSee™ IOL, were compared to those of a diffractive EDF, TECNIS Symfony™ IOL, of the same platform.

**Results:**

The refractive EDF IOL provides similar range of vision to the diffractive EDF IOL with the same distance, and similar intermediate and near sVA. The refractive EDF IOL provides the same tolerance to hyperopia as the diffractive EDF but more tolerance to myopia. Halo pictures and simulations showed that the refractive EDF provides comparable dysphotopsia profile to the monofocal IOL and better than the diffractive EDF.

**Conclusions:**

The results of this preclinical study in clinically relevant conditions show that the new refractive EDF IOL is expected to provide similar range of vision to the diffractive IOL of the same platform and higher tolerance to refractive errors. The refractive EDF provides a dysphotopsia profile that is better than the diffractive EDF and comparable to that of the monofocal IOL, also in the presence of residual refractive errors.

## Introduction

Intermediate vision plays a crucial role in daily activities. Its growing importance is mostly due to the rise in technology usage. Computers, tablets and smartphones have become essential tools for many people. As of 2021, approximately 60% of Americans aged 65 and above owned a smartphone, and 44% owned a tablet [1]. Additionally, the cataract population is experiencing a shift towards a more active lifestyle. Currently, even younger patients are opting for intraocular lens (IOL) implants, allowing them to continue driving, practicing sports, and, of course, use their smartphones and computers. In 2014, TECNIS Symfony (Johnson and Johnson Surgical Vision, Irvine, US) was introduced as the first extended depth of focus (EDF) IOL. This design was introduced to deliver a high-quality uninterrupted range of vision from far to near, maintaining better distance image quality than that provided by multifocal IOLs. In 2018, the American National of Standardization Institution (ANSI) created the EDF category by defining the criteria based on distance and intermediate vision, and defocus curve testing. Currently there are many EDF IOL options available for patients [2].

Although EDF IOLs provide a continuous range of vision, some models can be associated with reduced contrast sensitivity as compared to a monofocal IOL [3, 4] and higher levels of photic phenomena [5–7]. Enhanced monofocal IOLs were introduced to fill the gap between standard monofocal IOLs and EDF IOLs by creating a slightly extended depth of focus while maintaining all the benefits of a monofocal IOL such as low incidence of dysphotopsias and high distance image quality. A meta-analysis performed by Wan et al. [8] showed that the TECNIS Eyhance IOL, the first enhanced monofocal IOL available on the market, effectively improves intermediate vision compared to conventional monofocal IOLs. It also provided similar distance vision performance, contrast sensitivity and photic phenomena compared to conventional monofocal IOLs [8]. However, as described by Fernandez et al. [9] enhanced monofocal IOLs do not provide the range of vision required to satisfy the ANSI EDF criteria.

In this study, we introduce a new refractive EDF IOL that utilizes the same refractive technology as the enhanced monofocal IOL TECNIS Eyhance (Johnson and Johnson Surgical Vision, Irvine, US) [10], that is characterized by a continuous change in refractive power. The new IOL is designed to provide an even wider range of vision to qualify as an EDF IOL according to the ANSI criteria. Additionally, it is intended to increase the ease of use of EDF IOLs by maintaining the dysphotopsia profile of a monofocal IOL and increasing the tolerance to refractive errors for more predictable outcomes. The design concept as well as the simulated clinical performance of this new IOL design were evaluated using optical bench testing and computer simulations in clinically relevant conditions. The outcomes were compared to these of a diffractive EDF IOL of the same platform, the TECNIS Symfony IOL.

## Materials and methods

### IOL description

The new refractive EDF IOL is based on a continuous change in refractive power that distributes the light from far, through intermediate to near. The lens has the same overall dimensions and geometry as the all the lenses of the TECNIS 1-pc platform (Johnson and Johnson Surgical Vision, Irvine, US) as well as the same anterior surface designed to compensate for the average corneal spherical aberration (SA) [11].

**Fig. 1:**
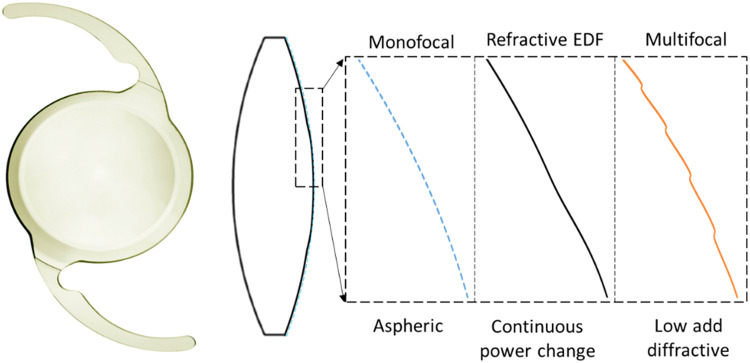
Front picture of the refractive EDF IOL (left) and illustration of the cross-section of the optics body (right). To illustrate differences between IOL designs, the posterior surface has been magnified for a monofocal IOL, the refractive EDF IOL and a low-add diffractive multifocal IOL.

The continuous power profile that characterizes the new refractive design was created by changing the curvature of the posterior surface of the lens from a base spherical design. Therefore, instead of refracting the light to a single focus like a monofocal IOL, the unique shape of the refractive EDF IOL refracts light to create an elongated focus and enables a continuous range of high-quality vision from far to near. Figure 1 illustrates the overall shape of the refractive EDF IOL and the modification with respect to the standard aspheric monofocal IOL and to a low-add (+2.75D) diffractive multifocal IOL. The change in curvature of the posterior surface of the lens has been magnified to illustrate the concept. A picture of the real lens is also included. As illustrated in Fig. 1 the refractive EDF IOL does not have sharp or abrupt changes in elevation, edges orsteps. The change in elevation occurs smoothly over a large distance whereas the multifocal IOL shows the abrupt steps that constitute the diffractive rings (Fig. 1).

This technology based on a continuous change in power was also used to design the TECNIS Eyhance IOL. The new refractive EDF IOL design introduced in this study makes use of the same principles of action with a completely different power profile to further increase the range of vision and provide functional near performance. As the TECNIS Eyhance IOL, the new refractive EDF IOL is not based on spherical aberration.

In this study, the new refractive EDF IOL was compared to the diffractive EDF IOL of the same platform, the TECNIS Symfony IOL (model ZXR00). TECNIS Symfony is a diffractive IOL designed to elongate the focus to provide a continuous range of high-quality vision, with the addition of the achromat technology to correct for longitudinal chromatic aberration and increase distance image quality [12].

Although it is approved in several regions, the new refractive EDF IOL is not approved in the US yet.

### IOL performance

#### Range of vision

Binocular visual acuity (sVA) was simulated using the MTFa measured in an eye model with an average corneal SA following the methodology described by Alarcon et al. [13] Measurements were collected in clinically relevant conditions, including white light for an average photopic pupil size of 3 mm from −2.5D to 1D of defocus in 0.5D steps.

Additionally, computer simulations using 46 realistic eye models were performed to evaluate the effect of corneal higher-order aberrations on far (0D), intermediate (−1.5D) and near (−2.5D) visual acuities. These eye models were also used to evaluate the effect of decentration. This methodology was presented by Weeber et al. [14] and it has been used to address the through focus performance of other IOL designs as well as the effect of decentration [10, 12].

#### Photic phenomena

To evaluate the dysphotopsia profile, halo pictures were measured using the GIT1 system [15]. The GIT1 system is an experimental device that consists of an eye model into which an IOL is loaded and then attached to a system of relay optics that allows subjects to “look through” the IOL and view a scene including any photic phenomena induced by the IOL or collects images using a camera in the subject’s plane. A previous study showed that the GIT1 system can be used to simulate photic phenomena induced by different IOL technologies in phakic eyes, providing a high correlation with the subjective perception of photic phenomena found in cataract patients implanted with the same IOLs [15]. Natural images in white light with a central glare source were collected with a fixed camera in the position of the subject using the GIT1 system for a 4 mm aperture to simulate mesopic light conditions.

Additionally, the dysphotopsia profile in the presence of refractive errors was evaluated using computer simulations of a point light source in polychromatic light conditions and 4 mm aperture using an average eye model based on the Liou–Brennan Eye model [16]. Simulations were performed inducing ±0.5D of defocus.

#### Tolerance to refractive errors

To evaluate the tolerance to residual defocus, computer simulated monocular VA (cVA) was calculated using the same 46 physiological eye models with realistic corneas and higher-order aberrations [14]. Simulations were performed in white light with 3 mm pupil to calculate the optical transfer function (OTF). From those simulations, visual acuity was calculated using the radial average weighted OTF (wOTF) to account for rotational asymmetries. Simulations were performed with the best correction in place and with spherical refractive error (±0.5D of defocus). This methodology was introduced by Alarcon et al. and has shown very high correlation with the clinical data (R^2^ = 0.97) [17].

## Results

**Fig. 2:**
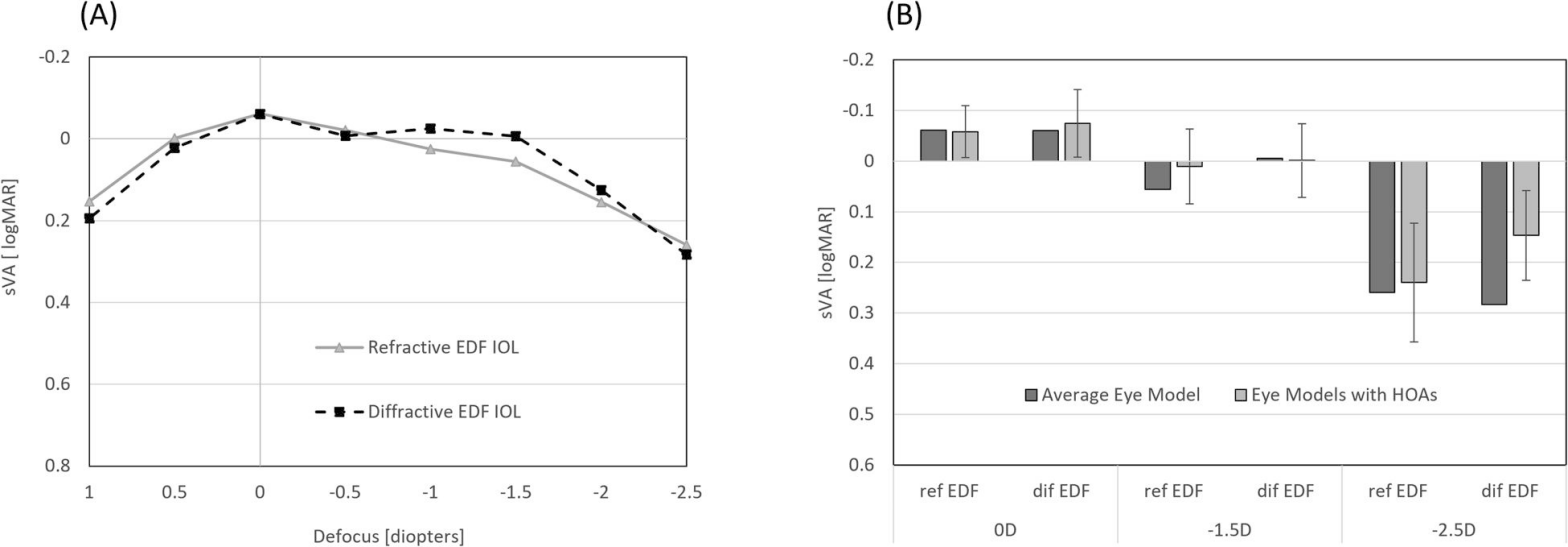
Simulated through focus visual acuity. **A** Simulated binocular visual acuity (sVA) calculated from optical bench measurements in an average physical eye model. **B** sVA measured in the average corneal eye model (Average Eye Model) and computer simulated in realistic eye models with varying corneal higher order aberrations (Eye models with HOAs) for far (0D), intermediate (−1.5D) and near (−2.5D) for the refractive (ref EDF) and the diffractive (dif EDF) EDF IOLs. Figures provide mean values ± standard deviation.

Figure 2A shows the sVA obtained for the refractive EDF IOL and the diffractive EDF IOL, the TECNIS Symfony IOL. The results show that the refractive EDF provides the same far and near VA compared to the diffractive EDF and a difference of 0.05 logMAR at intermediate. The range of vision where VA is equal or better than 0.20 logMAR was −2.2D for both the refractive EDF and the diffractive EDF IOLs.

Computer simulations show that the addition of higher order aberrations does not reduce the average performance at far (0D), intermediate (−1.5D) or near (−2.5D) of the refractive EDF IOL as compared to the average eye model measurements (Fig. 2B). Computer simulations also confirm the comparable distance performance at far and intermediate of the diffractive and the refractive EDF IOLs. In the presence of higher-order aberrations, computer simulations show better near VA for the diffractive EDF IOL than the sVA measured in the average eye model and the predicted near VA of the refractive EDF IOL. Computer simulations to evaluate the effect of decentration show that the simulated VA provided by the refractive EDF decreases by 0.03 and 0.04logMAR for 0.5 and 1 mm decentration, respectively, and by 0.05 and 0.09logMAR for the diffractive EDF IOL.

Additionally, computer simulations to evaluate the effect of ±0.5D of defocus on uncorrected distance sVA show that there is no difference between the refractive and diffractive EDF IOLs in the presence of hyperopia (96% and 100% of the eyes achieved 0.10logMAR or better in the presence of hyperopia with the refractive and diffractive EDF IOLs respectively). However, in the presence of myopia, the refractive EDF IOL results in 24% more eyes achieving monocular 0.10logMAR or better than in the diffractive EDF (96% of the eyes for the refractive EDF vs 72% for the diffractive EDF).

**Fig. 3:**
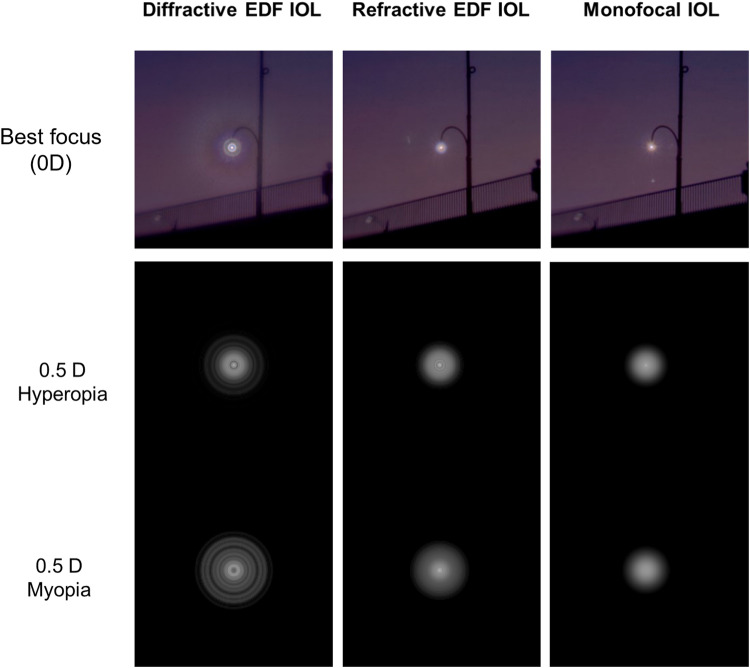
Halo measurements at best focus (0D) and simulations of a point light source under myopia and hyperopia.

To illustrate photic phenomena, Fig. 3 shows halo pictures of a central glare source with a natural background. These pictures were obtained with the refractive and diffractive EDF IOLs and the aspheric monofocal IOL, the TECNIS 1-pc Model ZCB00, of the same platform as a reference. These images show that the refractive EDF provides lower levels of dysphotopsia than the diffractive EDF IOL, and similar levels to the monofocal IOL. Simulations show that in the presence of ±0.5D of defocus, the refractive EDF IOL has the same halo performance as a monofocal IOL (Fig. 3).

## Discussion

This study introduces a new refractive EDF IOL and compares its performance to that of a diffractive EDF IOL of the same platform, the TECNIS Symfony IOL model ZXR00. The results of this study show that the new refractive EDF IOL provides a similar range of vision than the diffractive EDF IOL, −2.2D above 0.2 logMAR binocular VA, with same distance and comparable intermediate and near VA. Although the study is based on optical bench measurements and computer simulations, the results of these simulations have been shown to be well correlated with the clinical data of the TECNIS Symfony IOL [18]. For example, the binocular BCDVA, DCIVA and DCNVA of the TECNIS Symfony IOL reported in a clinical trial with 148 patients implanted was −0.05, 0.03 and 0.23 logMAR [6], respectively, which is in good agreement with the simulated VA provided in Fig. 2.

Additionally, the results of our study indicate that the new refractive EDF is expected to provide similar uncorrected distance vision to the diffractive EDF IOL in the presence of hyperopia and better VA and lower photic phenomena in the presence of myopia. Previous studies have shown the high tolerance to refractive errors of TECNIS Symfony IOL, providing high levels of UCDVA and patients satisfaction in the presence of small residual refractive errors [19]. Cochener 2017 showed that astigmatism up to 0.75D has a mild impact for far, intermediate and near uncorrected VA [20]. Therefore, it is expected that the new refractive EDF IOL will benefit from this high tolerance, increasing the ease-of-use of the lens and patient satisfaction [21]. Moreover, the high tolerance to myopic outcomes is an added benefit when monovision is targeted. TECNIS Symfony has been shown to provide good patient satisfaction, spectacle independence and low photic phenomena under monovision [19]. Based on the results of our study, we expect that the new refractive EDF IOL can be an even better option when targeting monovision.

Although better than a multifocal IOL, clinical results show that patients implanted with the TECNIS Symfony IOL can still report difficulty and some levels of bother with photic phenomena [18]. This is aligned with the results of our optical bench measurements that show a slight increase in the dysphotopsia profile of the TECNIS Symfony as compared to the monofocal IOL. To reduce the incidence of photic phenomena, the new refractive EDF IOL was designed to eliminate the most important sources of scatter. The design does not have rings, sharp changes in elevation, or zones with a constant add power. It is based on a continuous change in refractive power created by a smooth change in curvature of the posterior surface of the lens. This explains the results of the optical bench and simulations that show the monofocal-like dysphotopsia profile of the new refractive EDF design even in the presence of refractive errors (Fig. 3). This refractive technology was previously used in the design of the TECNIS Eyhance IOL [10], which has shown similar levels of dysphotopsia as a standard monofocal IOL [22, 23].

**Fig. 4:**
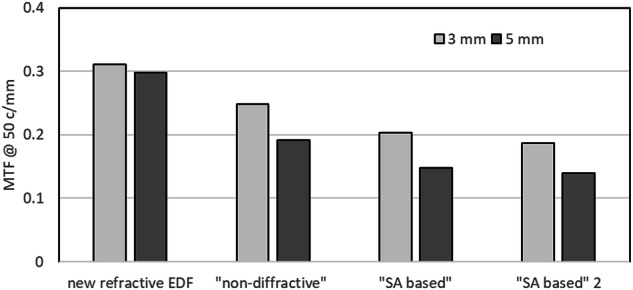
MTF measured at 3 and 5 mm pupil in an average corneal eye model in white light for the new refractive EDF IOL, an EDF IOL defined by manufacturer as “non-diffractive” and two IOLs designed to extend the depth of focus and defined by their manufacturers as based on Spherical Aberration (“SA based” and “SA based 2”).

Currently, there are other refractive or “non-diffractive” EDFs on the market, such as Vivity (Alcon Inc., US). Although this design increases the depth of focus as compared to the monofocal IOLs, it also results in a reduction in contrast sensitivity [3, 4] to the level of trifocal IOLs [24]. An estimation of the contrast sensitivity can be performed by comparing the MTF values measured in an optical bench using clinically relevant conditions [25]. Using this methodology, Fig. 4 shows that the new refractive EDF IOL provides an improvement in image contrast as compared to other refractive and non-diffractive EDF IOLs available on the market, with between 20 and 40% image contrast improvement at 3 mm pupil (photopic conditions) and between 36 and 53% improvement at 5 mm pupil (mesopic conditions). Moreover, the new refractive EDF IOL provides less pupil dependency when the pupil size changes from photopic to mesopic conditions than other EDF IOLs.

Contrary to diffractive IOLs that are known to modify chromatic aberrations [26], the new EDF design presented in this study demonstrated a refractive behavior, as its measured longitudinal chromatic aberration was practically constant for all pupil sizes, which was not the case for the “wavefront shaping” IOL [27]. It is important to note that although the new EDF design is purely refractive, the design is not based on SA. Contrary to designs based on SA, the new refractive EDF provides the same distance image quality for photopic and mesopic light conditions (Fig. 4).

This study introduces a new refractive EDF IOL designed to provide a continuous range of vision from far to near. Optical bench measurements and computer simulations show that the new refractive EDF IOL is expected to provide a similar range of vision as the diffractive TECNIS Symfony IOL, high tolerance to refractive errors, and a dysphotopsia profile similar to that of a monofocal IOL, even in the presence of refractive errors.

## Summary

### What was known before:

- Extended depth of focus IOLs provide larger depth of focus than monofocal IOLs.
- Extended depth of focus IOLs provide less side effects than multifocal IOLs.

### What this study adds:

- this study introduces a new refractive extended depth of focus IOL
- optical bench and simulations show that the new refractive extended depth of focus IOL is expected to provide the large range of vision of an extended depth of focus IOL with a dysphotopsia profile comparable to a monofocal IOL

## Data Availability

The datasets generated during and/or analyzed during the current study are not publicly available but are available from the corresponding author on reasonable request.
