# Correction: Optical and clinical simulated performance of a new refractive extended depth of focus intraocular lens

**DOI:** 10.1038/s41433-024-03423-4

**Published:** 2024-10-31

**Authors:** Aixa Alarcon, Antonio del Aguila Carrasco, Franck Gounou, Henk Weeber, Carmen Cánovas, Patricia Piers

**Affiliations:** Johnson and Johnson Vision Van Swietenlaan 5, Groningen, 9728 NX The Netherlands

**Keywords:** Outcomes research, Quality of life

Correction to: *Eye* 10.1038/s41433-024-03041-0, published online 05 April 2024

The ‘OBJECTIVES’ section of the abstract was corrected from “The purpose of this study is to evaluate the optical and expected clinical performance of a new refractive Extended Depth of Focus (EDF) intraocular lens (IOL) designed to maintain a monofocal-like dysphotopsia profile.” to “The purpose of this study is to evaluate the optical and expected clinical performance of a new refractive Extended Depth of Focus (EDF) intraocular lens (IOL), TECNIS PureSee™ IOL, designed to maintain a monofocal-like dysphotopsia profile.” Additionally, in the ‘METHODS’ section of the abstract, the last sentence was corrected from “The results of the refractive EDF were compared to those of a diffractive EDF of the same platform.” to “The results of the refractive EDF, TECNIS PureSee™ IOL, were compared to those of a diffractive EDF, TECNIS Symfony™ IOL, of the same platform.” The original article has been corrected.

